# Hybrid EEG-fNIRS phoneme classification based on imagined and perceived speech

**DOI:** 10.3389/fnrgo.2026.1696865

**Published:** 2026-02-10

**Authors:** Manuel Hons, Silvia Erika Kober, Selina Christin Wriessnegger, Guilherme Wood

**Affiliations:** 1 Department of Psychology, University of Graz, Graz, Austria; 2 Institute of Neural Engineering, Graz University of Technology, Graz, Austria; 3 BioTechMed-Graz, Graz, Austria

**Keywords:** Brain-computer-interface (BCI), classification, electroencephalography (EEG), functional near-infrared spectroscopy (fNIRS), hybrid, imagined speech, machine learning

## Abstract

**Introduction:**

Individuals affected by severe motor impairments often have no means of communicating with others. To build an intuitive speech prosthesis, imagined speech brain-computer interface research began to prosper with numerous studies attempting to classify imagined speech from brain signals. While unimodal neuroimaging techniques, such as electroencephalography (EEG) and functional near-infrared spectroscopy (fNIRS) have been widely used, multimodal approaches combining two or more of them remain scarce.

**Methods:**

In this study offline phoneme decoding based on hybrid EEG-fNIRS data was performed. Twenty-two right-handed participants performed imagined and perceived speech trials encompassing four phonemes /a/,/i/,/b/ and /k/. Features in the form of power spectral densities and mean hemoglobin concentration changes were extracted from EEG and fNIRS data, respectively. Features were ranked according to the mutual information criterion relative to the target vector, and the optimal number of features to include was determined through optimization via 10-fold cross-validation.

**Results:**

Hybrid classification yielded accuracy scores of 77.29% and 76.05% regarding imagined and perceived speech, respectively. In both conditions, hybrid and EEG-based classification performances did not differ significantly, while fNIRS based phoneme discrimination produced lower accuracies.

**Discussion:**

This study represents an innovative phoneme decoding attempt based on multimodal EEG-fNIRS data, both in terms of imagined speech and perception. Four-class imagined speech classification was primarily driven by EEG features yet outperformed comparable previous studies.

## 1 Introduction

Brain-Computer Interfaces (BCIs) are designed to enable individuals with motor impairments to control a certain device or software via brain activity. In the speech domain the primary objective of BCI research is the development of communicative prostheses for dysarthric individuals, such as people with amyotrophic lateral sclerosis or locked-in syndrome. P300-spellers (Farwell and Donchin, 1988) represent a popular variant of speech prostheses. The user is supposed to focus on a letter in a rectangular grid whose rows and columns get highlighted in a pre-defined or random order. Character selection is determined by the amplitude of the P300 component of the event related potential as a reaction to highlighting the row or column of interest. In a similar approach imagined movements are used to select characters partitioned into hexagonal fields (Blankertz et al., 2006). By imagining a hand movement an arrow is rotated. Imagining a foot movement selects a particular hexagon. The same procedure is applied to character selection from inside a hexagon. Eminently, both approaches can be time consuming and fatiguing as attention often must be maintained over a long period of time, especially when one wants to type a sentence or more. Both approaches lack intuitiveness, as the mental tasks that are used for character selection do not relate to the speech act itself. Furthermore, both approaches consist of a visual paradigm, where the user must interact with what is displayed on a computer monitor. Therefore, such systems clearly are impractical for cases where motor impairments additionally affect visual perception. For the above reasons imagined speech has recently risen to popularity as a potentially discriminable BCI-task. Imagined speech refers to the act of mentally ‘speaking' without moving the articulators. Several levels of imagined speech prompts have been subjected to classification, such as phonemes (Matsumoto and Hori, 2014), syllables (Jahangiri and Sepulveda, 2019), words (Rezazadeh Sereshkeh et al., 2017) and word combinations (Cooney et al., 2022).

Most of the literature engaging in imagined speech classification relies on unimodal electroencephalographic (EEG) brain signal acquisition. Thereby the prefrontal cortex, the superior temporal gyrus, the temporo-parietal junction, the primary motor cortex and the inferior frontal cortex were found to elicit electrophysiological signals that discriminate well between imagined speech entities (Nguyen et al., 2018; Rezazadeh Sereshkeh et al., 2019b; Preedapirat and Wongsawat, 2022; Lu et al., 2023). These areas are strongly coherent with those generally associated with speech imagery, but also production (Pei et al., 2011; Tian and Poeppel, 2013). Further, in terms of the spectral content of EEG signals, beta and gamma frequency bands are attributed higher discriminative capabilities compared to lower frequency bands (Jahangiri and Sepulveda, 2019; Rezazadeh Sereshkeh et al., 2019b; Preedapirat and Wongsawat, 2022). Despite the emerging knowledge about the discriminative capabilities of features in the imagined speech realm, classification accuracies often either fall below the recommended threshold for practical BCI use of 70% (Kübler et al., 2006; DaSalla et al., 2009; Cooney et al., 2022), or are produced via binary classifications in a multiclass problem (Matsumoto and Hori, 2014; Chengaiyan et al., 2020; Sarmiento et al., 2021).

In the urge to improve multiclass decoding performance, leveraging signals of multiple neuroimaging methods appeared to be successful in other research fields, such as motor imagery classification (Fazli et al., 2012; Khan et al., 2014; Chiarelli et al., 2018; Shin et al., 2018). Combining EEG and functional near-infrared spectroscopy is a popular approach to such multimodal measurements (Li et al., 2017, 2022; Hong et al., 2018). Since EEG is constrained to electrical signal measurement and highly susceptible to motion artifacts (Liu et al., 2021), fNIRS represents a promising addition in terms of signal acquisition. fNIRS is an optical imaging method that measures signals from hemodynamic sources by emitting and detecting near-infrared light and hence less susceptible to motion artifacts (Quaresima and Ferrari, 2019; Liu et al., 2021). One drawback of fNIRS, however, is the low temporal resolution: since the fNIRS signal strongly depends on the neurovascular bloodflow, the event-related fNIRS response peaks at ~4.6 seconds and lasts for ~15-20 seconds in total (Huppert et al., 2006). This constitutes a major problem in terms of online classification as it is not practical to depend on a 20-second classification window (Liu et al., 2021). Therefore, when keeping a potential online-transfer in mind, intelligently combining the strengths of both modalities and finding a compromise between trial length and the preservation of the most important signal qualities (Rezazadeh Sereshkeh et al., 2019b; Cooney et al., 2022; Fan et al., 2024) is pivotal. The subsequent paragraph addresses the issue of limited temporal resolution in fNIRS by proposing the extraction of features that quantify the goodness of fit between the measured signal and the canonical hemodynamic response function (HRF).

Regarding imagined speech classification, only few studies acquired metabolic, let alone, multimodal brain signals. In one multimodal study (Rezazadeh Sereshkeh et al., 2019b), for instance, the discrimination between “yes”, “no” and rest trials from EEG and fNIRS data was evaluated. The hybrid classification approach incorporating two different signal sources yielded an improved accuracy of 70.45% compared to unimodal EEG and fNIRS protocols with 63.76% and 63.64%, respectively. Although to a lesser extent, a demonstration of this synergistic effect grounded on multimodal EEG-fNIRS based classification also exists with respect to word combinations (Cooney et al., 2022). The authors reported a hybrid classification accuracy of 32.21%, an EEG-based accuracy of 31.55%, and an fNIRS-based accuracy of 31.32% for four different word-combinations. Note that the authors incorporated 2 s trials and extracted a maximum of 50 trials per participants. However, this is not the only instance of a classification paradigm with short fNIRS trials (Harrivel et al., 2016; Chiarelli et al., 2018). In a classification study by Chiarelli et al. (2018) a hybrid EEG-fNIRS BCI was developed to discriminate between left and right hand motor imagery on the basis of 5 s trials. Unimodal EEG and fNIRS classifications yielded 73.38% and 71.92%, respectively. Importantly, hybrid classification improvement was registered upon combining both datasets, culminating in a decoding accuracy of 83.28%. Beyond classification studies, a large body of fNIRS literature exists that demonstrates the feasibility of rapid and predominantly event-related designs (Plichta et al., 2007; Schaeffer et al., 2014; Harrivel et al., 2016; Kober and Wood, 2017). Thereof, many perform analyses based on beta coefficients, indicating the goodness of fit between a certain fNIRS signal and the canonical HRF, rather than raw hemoglobin signal changes (Plichta et al., 2007; Harrivel et al., 2016; Kober and Wood, 2017). This appears to be especially useful in rapid designs, as task-related response contaminations of preceding trials are accounted for by incorporating a model of the canonical HRF. Therefore, extracted beta coefficients might be more stable across trials, which might benefit classification procedures.

Despite the potential benefits of acquiring signals from multiple neuroimaging sources, the complexity of the data inevitably increases. To deal with the inflation of noise and to prevent overfitting (Ying, 2019), reducing the dimensionality of the feature set by eliminating uninformative features can be useful (Mwangi et al., 2014). Various feature selection techniques have been employed in the existing literature, such as genetic algorithms (Ramos et al., 2016), recursive feature elimination strategies (Shen et al., 2007), and feature selection strategies based on *t*-values (Li et al., 2017) and mutual information scores (Deligani et al., 2021), both of which indicate the discriminative capabilities of data channels with respect to the classes.

Imagined speech generally is considered to evoke subtler differences in neural signatures and consequently lower classification scores than its pendants, perceived speech and speech production (Martin et al., 2016; Sharon et al., 2020). Consequently, some researchers either exclusively classified perceived speech (Kim et al., 2014; Moinnereau et al., 2018) or incorporated it as an additional condition alongside imagined speech (Martin et al., 2016; Sharon et al., 2020; Cooney et al., 2022). A recent fMRI study showed that perceived speech elicits neural activity in areas strongly overlapping with those involved in imagined speech (Lu et al., 2023): the bilateral superior temporal gyri, the right temporal pole, the right precentral gyrus and supplementary motor areas. Further, opposed to imagined speech, people partaking in a perceived speech task are less susceptible to myogenic artifacts (Orpella et al., 2022), making it a useful low-noise reference condition for speech imagery classification paradigms.

Since most studies in this field perform binary classifications, even in multiclass-problems, the present work investigates the decoding performance of a four-class phoneme classification problem. Participants completed both an imagined speech and a perceived speech task. We incorporated a feature selection mechanism based on the mutual information criterion to reduce the complexity of the data and to improve classification results. In contrast to most existing studies, we classify imagined speech as well as perceived speech by leveraging hybrid signals based on EEG and fNIRS. While the present study engages in the classification of both imagined and perceived speech, the focus lies on the former.

## 2 Materials and methods

### 2.1 Participants

Twenty-seven right-handed participants (14 women and 13 men) were recruited to participate in the study. Due to technical issues during measurements, three participants had to be excluded. Further, the data of two participants were excluded due to excessive artifact and noise corruption. This resulted in a final sample of 22 participants between the ages of 19 and 37 (*M* = 25.41, *SD* = 4.02). All participants were native German speakers, showed no hearing problems and had normal or corrected-to-normal vision. None of the participants reported medical, psychiatric or neurological disorders that could influence the central nervous system, such as metabolic diseases, major depression, epilepsy etc. Further, participants reported no medication that could influence the central nervous system. Recruiting was carried out through university-wide mailing lists, social media and the personal circle of acquaintances. Participation compensation consisted of either 28 Euros (8 Euros per hour) or course credit for psychology students. In addition, participants were given the opportunity to receive feedback on the classification performance regarding their own data. Two participants decided to get such feedback. Prior to the study, all participants provided written informed consent. This study was approved by the ethics committee of the University of Graz, Austria (GZ 39/57/63 ex 2021/22) and is in accordance with the ethical principles of the Declaration of Helsinki.

### 2.2 Material

#### 2.2.1 Questionnaires

A COVID-19 questionnaire and a standard socio-demographic questionnaire, assessing age, gender, occupation, educational level etc., were administered. Additionally, participants were asked to complete the German version of Vividness of Movement Imagery Questionnaire 2 (Roberts et al., 2008; Dahm et al., 2019) and the Varieties of Inner Speech Questionnaire – Revised (Alderson-Day et al., 2018). Note that these two questionnaires were not subjected to the analyses of the current study, as they were assessed as part of a larger investigation.

#### 2.2.2 Phonemes

The phonemes of interest were /a/, /i/, /b/ and /k/. This choice was based on the articulatory differences between these phonemes. /b/ is defined as lax, voiced and bilabial in terms of production, whereas /k/ is a tense, voiceless, velar consonant (Keating, 1984). In terms of the selected vowels, /i/ is referred to as a closed front vowel due to the high and frontal position of the tongue, while /a/ is an open central vowel as the tongue adopts a low and central position during articulation (Tronka, 2004). Audio stimuli were recorded by the first author. Recorded audio signals were digitally manipulated so that each one had its fundamental frequency in the gender-ambiguous range of 140-170 Hz (Biemans, 2000), except for the /k/ phoneme audio, since /k/ does not exhibit formants or other representative frequencies, due to its voicelessness. Here the audio stimulus was modulated by ear until it sounded gender-ambiguous.

### 2.3 Procedure

**Figure 1:**
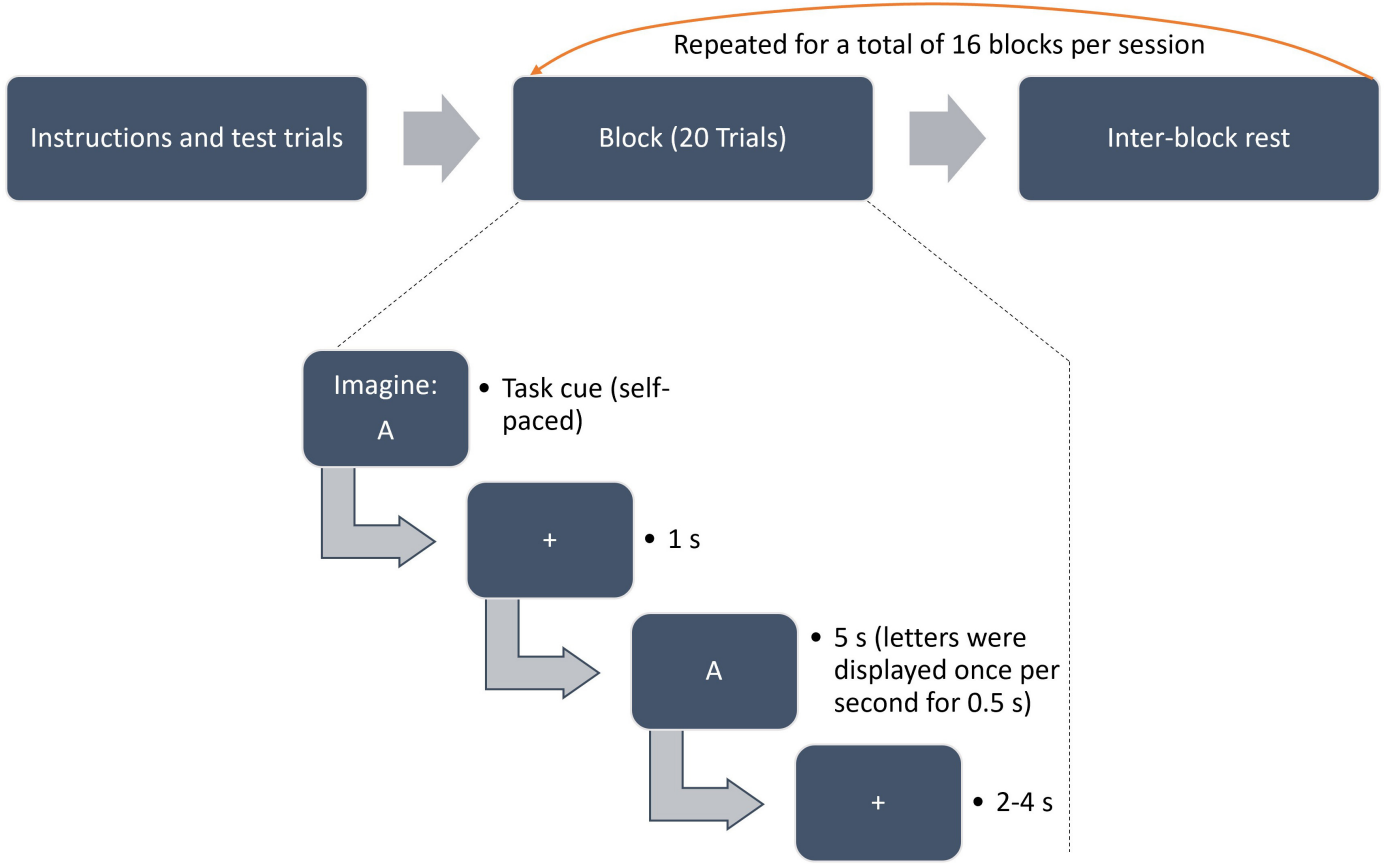
Schematic illustration of the experimental paradigm.

All participants attended two sessions per 1 h and 45 mins (3.5 h in total) on two separate days. They were seated in a comfortable armchair approximately 100 cm in front of a 24.5-inch computer screen. Before the actual experiment began, instructions were displayed on the monitor and participants completed 2 test trials for each condition: Imagined and perceived speech. During this phase, participants heard the audio recordings of all phonemes. They were then asked if they had any questions and had the opportunity to ask for additional test trials. Each participant completed both task conditions. In the perception condition, one of the four phonemes was presented audio-visually once per second for5 seconds, which represented one trial. The auditory presentation of phonemes was carried out via near field studio monitors (Presonus Eris 4.5) at a constant volume of approximately 75 db at the position of the participants. In the imagination condition, phonemes were only presented visually. A visual presentation manner was chosen instead of auditory stimuli in order to prevent neural traces of the stimuli in auditory brain areas as they are considered to be involved in the imagination of speech (Panachakel and Ramakrishnan, 2021; Lu et al., 2023). Participants had to imagine speaking the respective phoneme at the same beat as the visual presentation indicated, i.e., once per second for 5 seconds. The selection of relatively short trial periods was inspired by related previous studies (Chiarelli et al., 2018; Cooney et al., 2022; Guo and Chen, 2023), which demonstrated substantial classification accuracies despite having short fNIRS trials. Furthermore, a test measurement showed clear oxy-hemoglobin peaks within the averaged 5s trial period which is in line with previous findings (Huppert et al., 2006). Inter-trial rests of randomized duration between 2 and 4 seconds were implemented. Each block consisted of 20 trials presenting one phoneme. Each experimental session consisted of 16 pseudo-randomly occurring blocks (2 repetitions ^*^ 2 conditions ^*^ 4 phonemes) and lasted for approximately 50 minutes. Between blocks a break of at least 15 s was incorporated (participants could start each upcoming block after 15 s themselves by pressing a button). Having two blocks with 20 trials each per session for every phoneme-task combination, this resulted in a total of 80 trials per combination, of which there are: Perception /a/, Perception /i/, Perception /b/, Perception /k/, Imagination /a/, Imagination /i/, Imagination /b/ and Imagination /k/. The starting phoneme-task combination was balanced across participants. The second session then started off with the same phoneme but the other task. The experimental procedure is visually displayed in Figure 1. The entire paradigm was run in PsychoPy2 (version 1.85).

### 2.4 Data Acquisition

EEG, fNIRS, EMG, EOG, Respiration and ECG were recorded concurrently. Respiration and ECG signals were measured preemptively in case artifact correction based on short distance fNIRS channels did not work as intended. Since this was not the case, both measures were not subjected to the signal-processing or analyses. Since both the EEG and fNIRS recording software were running on the same computer, we used a single physical parallel port cable to send markers to both programs simultaneously, and synchronized the recordings based on these markers at a later step.

#### 2.4.1 EEG

**Figure 2:**
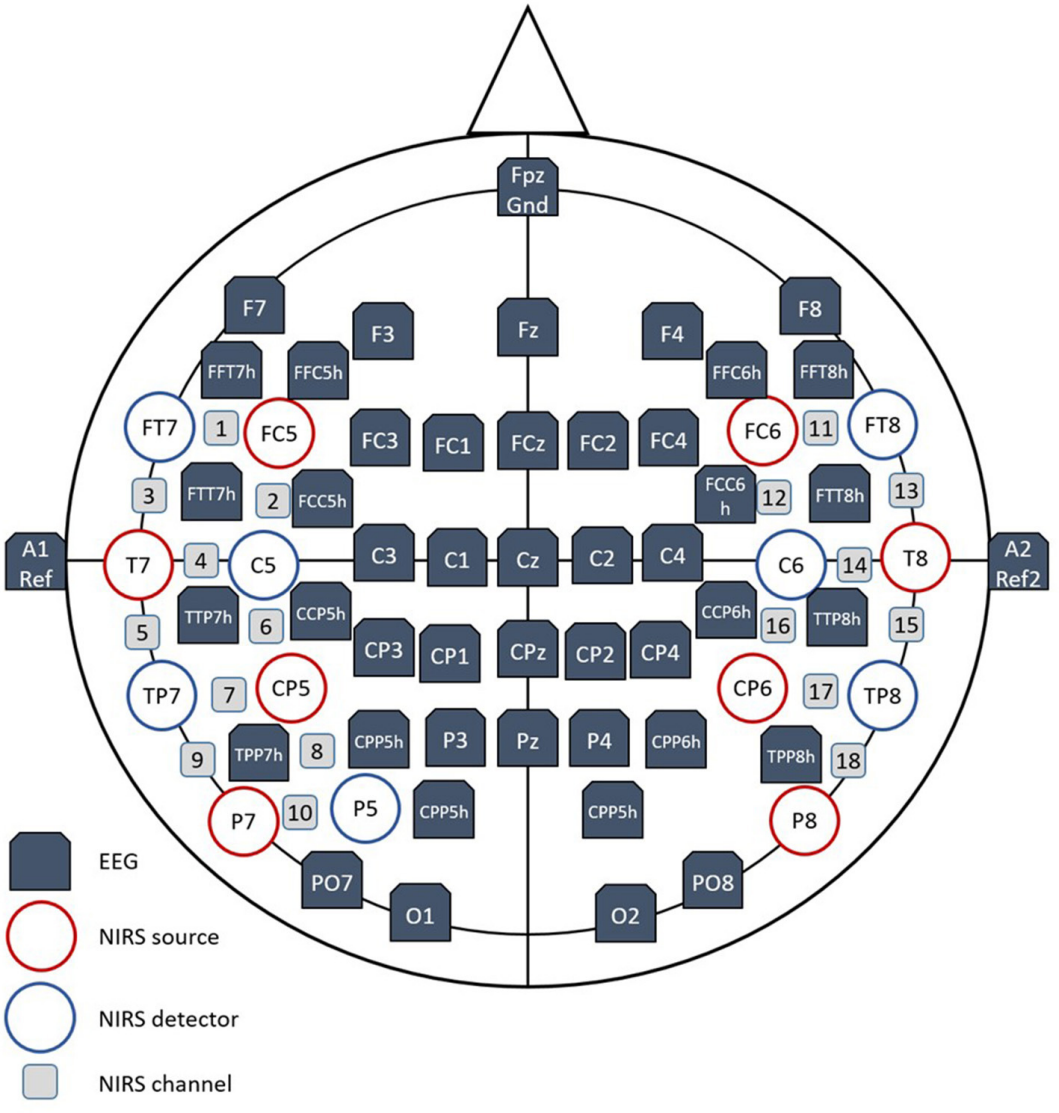
EEG-fNIRS montage set up.

EEG was recorded from 45 electrodes positioned across the whole head. Electrode positions conform to the international 10-5 system to facilitate electrode placement around temporal fNIRS optodes and to allow for an even distribution of electrodes across all cortical regions (Figure 2). EEG was measured using actiCAP active wet electrodes (Brain Products GmbH), a BrainAmp EEG amplifier (Brain Products GmbH) and the accompanying recording software BrainVision Recorder (version 1.21) at a sampling rate of 500 Hz. The left mastoid was used as the reference electrode. However, signals were re-referenced to the average signal of the left and right mastoid at a later stage. The ground electrode was positioned at Fpz. Electrooculogram (EOG) was obtained by applying three electrodes in total. One measured vertical eye movement and blinking and was placed approximately 1 cm above the nasion. The remaining two electrodes were placed at the lateral corners of the eyes to measure horizontal eye movements. In addition, cervical electromyogram was recorded by using two electrodes. One was placed on the right musculus sternocleidomastoideus and one was placed at the right side of the prominentia laryngea. However, EMG signals were not used in the current study as a visual inspection approach was adopted to account for muscular artifacts. Electrode impedances were targeted to be kept below 20 and 50 kΩ for EEG and EOG/EMG, respectively.

#### 2.4.2 fNIRS

fNIRS data were acquired using the NIRSport2 system (NIRx Medical Technologies, Germany) and the associated recording software Aurora fNIRS (version 1.4, NIRx Medical Technologies, Germany) at a sampling rate of 10.17 Hz. In this study, eight emitter- and seven detector optodes were arranged into two rectangular grids, one on each hemisphere (Figure 2). This arrangement resulted in 18 long-distance fNIRS channels with an emitter-detector distance of 30 mm. Additionally, eight short-distance detectors with an emitter-detector distance of 8 mm were incorporated into this setup to measure and correct for the physiological activity in the extra-cerebral tissue. Each source optode emitted light at wavelengths 760 nm and 850 nm.

### 2.5 Data Preprocessing

**Figure 3:**
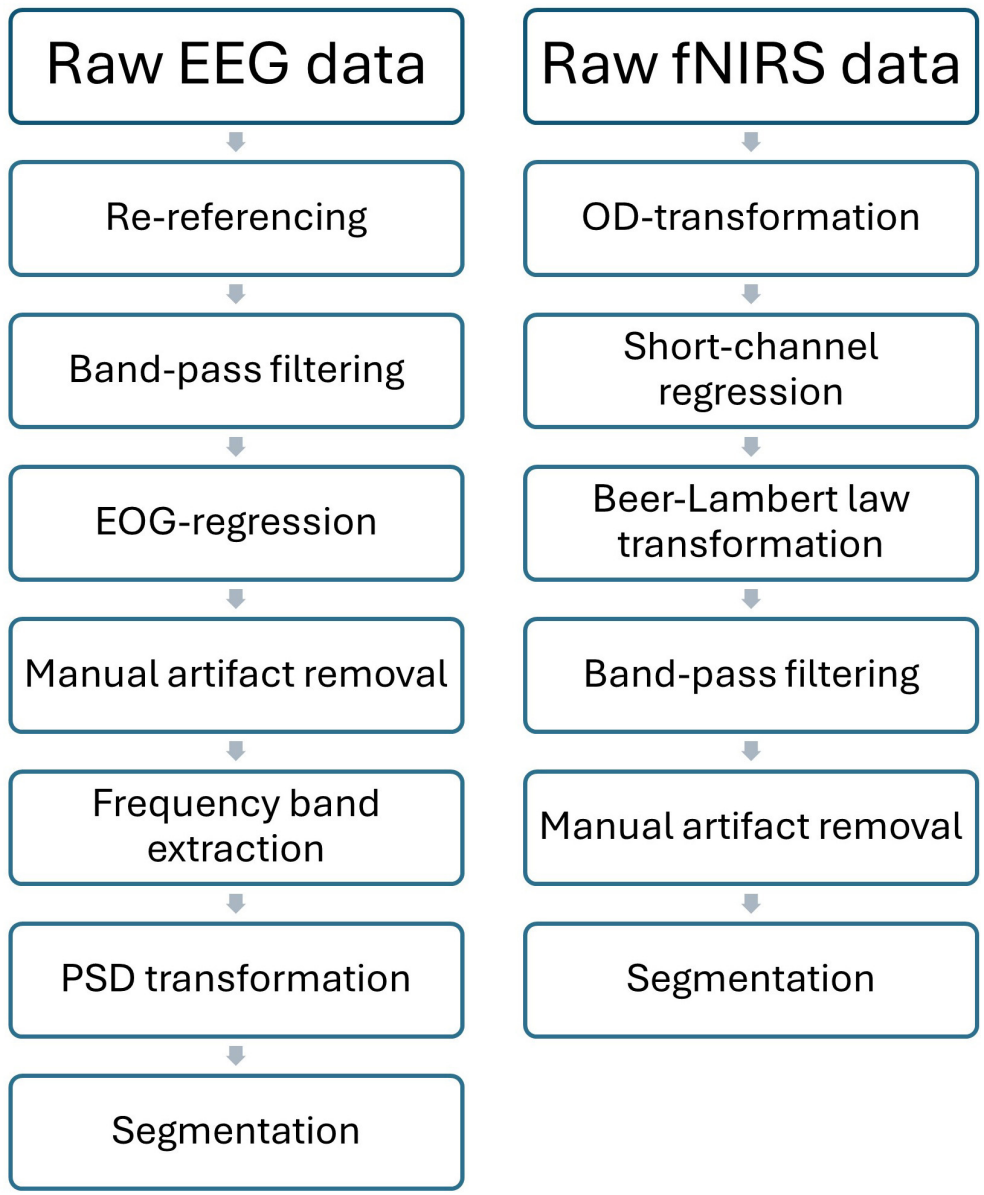
Pre-processing pipeline regarding EEG- and fNIRS-data.

All preprocessing steps were performed within Python 3.10 using the MNE-Python (version 1.8, Gramfort et al., 2013) and the MNE-NIRS (version 0.7, Luke et al., 2021) libraries for EEG and fNIRS, respectively. The EEG- and fNIRS-preprocessing-pipelines are depicted in Figure 3.

#### 2.5.1 EEG

During preprocessing, EEG data were re-referenced to the mean of the bilateral mastoid reference signals. A high-pass filter at 1 Hz, a low-pass filter at 70 Hz and a notch filter at 50 Hz were then applied to the data. Subsequently, EOG artifacts were corrected via regression (Gratton et al., 1983) by leveraging the EOG signals as reference signals. While most EEG research relies on some form of automatic artifact detection (Islam et al., 2016) which often require the definition of constant signal thresholds, we prefer manual artifact identification, as constant thresholds often neglect inter-individual variation (Jas et al., 2017). Therefore, artifact corrupted trials and noisy channels were then flagged for removal by visual inspection while taking established signal thresholds into account (Jas et al., 2017; Zhang et al., 2024). For the EEG data, artifact detection was focused on high-frequency motion artifacts, low-frequency ocular artifacts which would coincide with deflections in the EOG signals, as well as sudden spikes showing a voltage surpassing the threshold of ~200 μV or dramatic peak-to-peak differences of ~100 μV. The EEG data were then filtered according to the frequency bands of interest and transformed into power spectral density values (see Section 2.6). Finally, the EEG data were segmented into 5-second trial epochs, which were baseline-corrected by subtracting the mean of the 1-second baseline interval preceding each trial.

#### 2.5.2 fNIRS

First, raw fNIRS data were converted to optical density. Short channel regression was then applied to the optical density data (Fabbri et al., 2004; Scholkmann et al., 2014). This method is used to remove the influence of superficial tissue layers on the cerebral fNIRS signals by subtracting weighted signals originating from short distance channels from the fNIRS signal of interest. Corrected optical density data were then converted to concentration changes of oxy-hemoglobin (HbO) and deoxy-hemoglobin (HbR) using the modified Beer-Lambert law with a differential pathlength factor of 6.0 (Duncan et al., 1995). fNIRS data were high-pass filtered at 0.01 Hz and low-pass filtered at 0.7 Hz. In line with the best practice guidelines stated by Yücel et al. (2021) we manually removed trials corrupted by motion artifacts in the form of spikes or baseline shifts. In contrast to EEG literature, no established statistical thresholding recommendations exist with respect to motion artifact detection. The fNIRS signals were then segmented into 5-second trial epochs, which were then baseline corrected by subtracting the mean of the 1-second baseline interval preceding each trial.

#### 2.5.3 Numbers of trials

The remaining numbers of trials for each participant are displayed in Table 1. After manually removing EEG and fNIRS trials contaminated by artifacts, between 252 and 316 of the original 320 imagined speech trials remained per participant. Concerning the perceived speech task condition, between 255 and 318 trials were kept. Out of a maximum of 80 trials per class, participant 5 completed 58 usable trials in the ‘Perception /i/' condition, while participant 24 completed 59 usable trials in both the ‘Imagination /k/' and the ‘Perception /a/' phoneme-task-combination. All remaining participants showed at least 60 remaining trials for each phoneme-task-combination. Concerning the combined data of all participants, datasets with 6,379 and 6,330 trials were obtained (7,040 recorded trials minus manually removed artifact contaminated trials) for the phoneme perception and the phoneme imagination condition, respectively.

**Table 1:** Remaining trials after artifact correction.

**Participant**	**/a/**	**/i/**	**/b/**	**/k/**	**/a/**	**/i/**	**/b/**	**/k/**
P03	77	79	78	71	77	77	77	80
P04	77	76	75	73	75	78	75	79
P05	71	65	68	69	69	58	70	65
P06	76	77	74	67	77	68	71	73
P07	68	72	70	72	70	68	69	70
P08	73	74	75	69	71	75	72	78
P10	61	65	62	64	65	61	66	67
P11	74	79	75	78	75	76	75	75
P12	68	70	70	67	65	60	66	66
P13	78	80	76	75	78	78	78	66
P14	69	77	75	73	78	72	76	78
P15	73	70	69	68	68	67	71	64
P16	78	80	79	79	80	79	79	80
P17	76	77	76	72	74	70	74	72
P18	74	72	74	75	75	75	74	72
P19	62	64	78	71	74	64	73	73
P20	69	71	71	70	73	69	75	74
P21	79	80	79	77	80	79	79	78
P22	73	75	76	74	69	75	70	73
P24	67	64	72	59	59	65	66	65
P26	70	72	69	70	69	64	66	67
P27	71	73	73	76	78	74	71	71
Σ	1584	1612	1614	1569	1599	1552	1593	1586

### 2.6 Feature Selection

Regarding EEG, mean power spectral densities (PSDs) were calculated for a priori defined frequency ranges for each trial. These frequency ranges were 8-12 Hz (alpha band), 12-30 Hz (beta band) and 30-70 Hz (gamma band). This choice was based on evidence describing higher frequency bands as more discriminative with respect to an imagined speech classification problem (Jahangiri and Sepulveda, 2019; Rezazadeh Sereshkeh et al., 2019b; Preedapirat and Wongsawat, 2022). This resulted in a total of 135 features per trial (45 channels ^*^ 3 frequency bands) assuming that no channels were excluded.

From fNIRS trials, the mean HbO and HbR values were extracted for three different time windows (Hosni et al., 2022): 0-1 s, 1-3 s and 3-5 s after trial start. This was done to capture the early temporal dynamics of the hemodynamic response. In the present study, 108 features (18 channels ^*^ 2 chromophores ^*^ 3 time windows) were extracted for each trial, assuming that no channels were excluded. Additionally, in a second leg of fNIRS data analysis, we extracted beta coefficients indicating the goodness of fit between the raw fNIRS trial signals and the corresponding canonical HRF signals (SPM type). For each trial a linear regression was performed using the empirical fNIRS signal as a predictor and the data of the canonical HRF model as a criterion. Standardized regression coefficients (beta coefficients) were extracted and used as features for classification. This resulted in a set of 36 features (18 channels ^*^ 2 chromophores) provided that no channels were excluded. To provide clarity for the reader, throughout the rest of this manuscript, “fNIRS features” refer to the standard fNIRS features (mean hemoglobin changes of different time windows) and “beta features” refer to beta power features in the EEG domain, while “beta coefficients” are always explicitly mentioned when referring to fNIRS features based on beta coefficients.

EEG and fNIRS data were combined into hybrid datasets of 243 features. Thus, each trial was represented by one row in the feature matrix, comprising information from both the EEG and fNIRS modality. Note that unimodal datasets were retained, since the following steps were also applied to them. Subsequently, all data were z-transformed, i.e., data were centered on a mean of 0 with a standard deviation of 1. The features to recruit for classification were selected by means of the mutual information criterion (MI). MI is an information theoretic measure able to detect bivariate dependencies which do not necessarily manifest in covariance-based methods such as correlation analyses. MI equals 0 if two variables are independent, whereas higher values reflect stronger dependencies. For more information on MI please refer to Kraskov et al. (2004). More specifically, the dependencies between the categorical target vector and each feature vector produced an MI value. These are used to rank features according to their expected discriminability, as indicated by the MI. Note that this procedure was consistently only employed to the training portion of the dataset in order to avoid spillover effects. The dimensions of the underlying training datasets varied, depending on the number of trials rejected, with a maximum number of datapoints being 256 – 80% of the maximum number of trials per subject and class, 320.

### 2.7 Classification

Datasets were randomly split into training and test set using an 80/20 ratio. Stratification was employed to preserve relative class frequencies in both datasets. The identical train-test-splitting approach was used for both intra-subjects and cross-subjects classification. On an individual subject basis, this resulted in test set sizes varying from 51 to a maximum of 64 instances, with single classes exhibiting between 12 and 16 instances. Training set sizes ranged between 201 and 254 instances.

Classification was performed within Python 3.10. using the scikit-learn library (version 1.5.2, Pedregosa et al., 2011). A multilayer perceptron (MLP) served as the classifier. MLPs belong to the family of artificial neural networks (ANNs) and consist of multiple layers which in turn consist of multiple neurons. Superficially, the activation value of each neuron is determined by weights and a bias. The weights determine the relative relevance of each input, while the bias shifts the activation function along the X-axis, which influences the ‘excitability' of the corresponding neuron. When a training vector of feature values is put in, the network calculates the cost of the entire network by a predetermined cost function. By employing gradient descent, adjustments for all weights and biases with respect to minimizing the cost function are calculated. The adjustments are then applied by backpropagating through the entire network. This process is often referred to as ‘learning'. For a more detailed description of MLPs refer to Gardner and Dorling (1998). In this study, the MLP consisted of two layers with 200 and 20 neurons, respectively. The rectified linear unit function (ReLU) served as the activation function for each neuron. The L2-norm cost function was used for overall cost calculation.

Classification was conducted based on hybrid, as well as unimodal EEG- and unimodal fNIRS-data with respect to both conditions, imagined and perceived speech. Accuracy scores functioned as the primary evaluation metric. The corresponding confusion matrices, as well as precision, recall and F1-scores (the harmonized mean of precision and recall) are reported for the main intra-subject classification protocols (hybrid, EEG and fNIRS for both conditions). Classification procedures were not only performed on a single-subject basis, but also on concatenated data of all 22 participants. This was done using a purposely coarse concatenation without explicitly addressing inter-subject variability, as we sought to conduct an exploratory investigation of the feasibility of cross-subjects classification based on mere merging of individual feature matrices. Since cross-subjects classifications encompassed single classifications across the concatenated data of all participants, no means or standard deviations could be reported here. The cross-subject classification approach entailed the imputation of missing values caused by the elimination of electrode signals with bad data quality. This was performed by filling missing values with the electrode means of the training set. To avoid overfitting, we used a constant value to ensure the imputed information contained no artificial discriminatory information, which could have been potentially introduced by other imputation methods, such as regression. Further, to avoid introducing noise, we did not choose an arbitrary value but the mean, which is more representative of each feature's value range. However, the drawback of this method is that MI values of features with a lot of missing information shrink, due to the increased proportion of non-discriminative data. Supportingly, Shadbahr et al. (2023) found that mean imputation performed on par with other, multiple imputation methods in terms of classification performance. Both the cross-subjects and the individual classifications were conducted separately for the imagined speech and the perceived speech condition. To assess whether the mutual information based feature selection technique was beneficial, a baseline in the form of classification based on *k* features for each all *k* = 1, 2... 99, 100 is incorporated. Accuracy scores are reported for all classification procedures.

### 2.8 Hyperparameter optimization

All hyperparameters were optimized on the training set only. For each classification protocol, the optimal number of features was determined by calculating the average accuracy score produced by 5-fold cross-validation (CV) on the training set for *j* = 20, 25…65, 70 features with the highest MI score. Additionally, the L2 regularization parameter was adjusted. Out of the set of α = 10^−4^, 0.2, 0.4, 0.6, 0.8, 1, the regularization parameter with the highest CV-accuracy was selected for each classification protocol independently. Instead of including α = 0, which performs no regularization, we included the standard value for MLPs in scikit-learn, α = 10^−4^. Specifically, for each combination of *j* and α, CV was performed for all subjects and all three modalities (hybrid, EEG, fNIRS) with the resulting average CV-accuracies being stored. For each modality, the α with the largest average CV-accuracy was selected. Then, for each modality, α and subject, the *j* with the largest average CV-accuracy was selected. Note that α was only optimized on the intra-subject level of classifications and not on the cross-subjects level, as we wanted to ensure comparability between the two levels. Regarding the imagined speech condition, α-values of 10^−4^, 0.4, and 0.2 were selected for the hybrid, EEG, and fNIRS protocol, respectively, whereas in the perceived speech condition, α-values of 10^−4^, 10^−4^, and 0.4 were selected for the hybrid, EEG, and fNIRS protocol, respectively. Cross-language classification, classification using fNIRS beta coefficients and classifications based on *k* = 1, 2 … 99, 100 features were performed using the α-values extracted from the intra-subject protocols.

### 2.9 Statistical analysis

In order to test the significance of classification accuracy differences, we performed dependent t-tests with Bonferroni-Holm correction for multiple comparisons. Furthermore, to assess whether classification accuracies differ significantly from the chance level (25%), we applied a bootstrapping procedure with 10000 iterations.

## 3 Results

**Figure 4:**
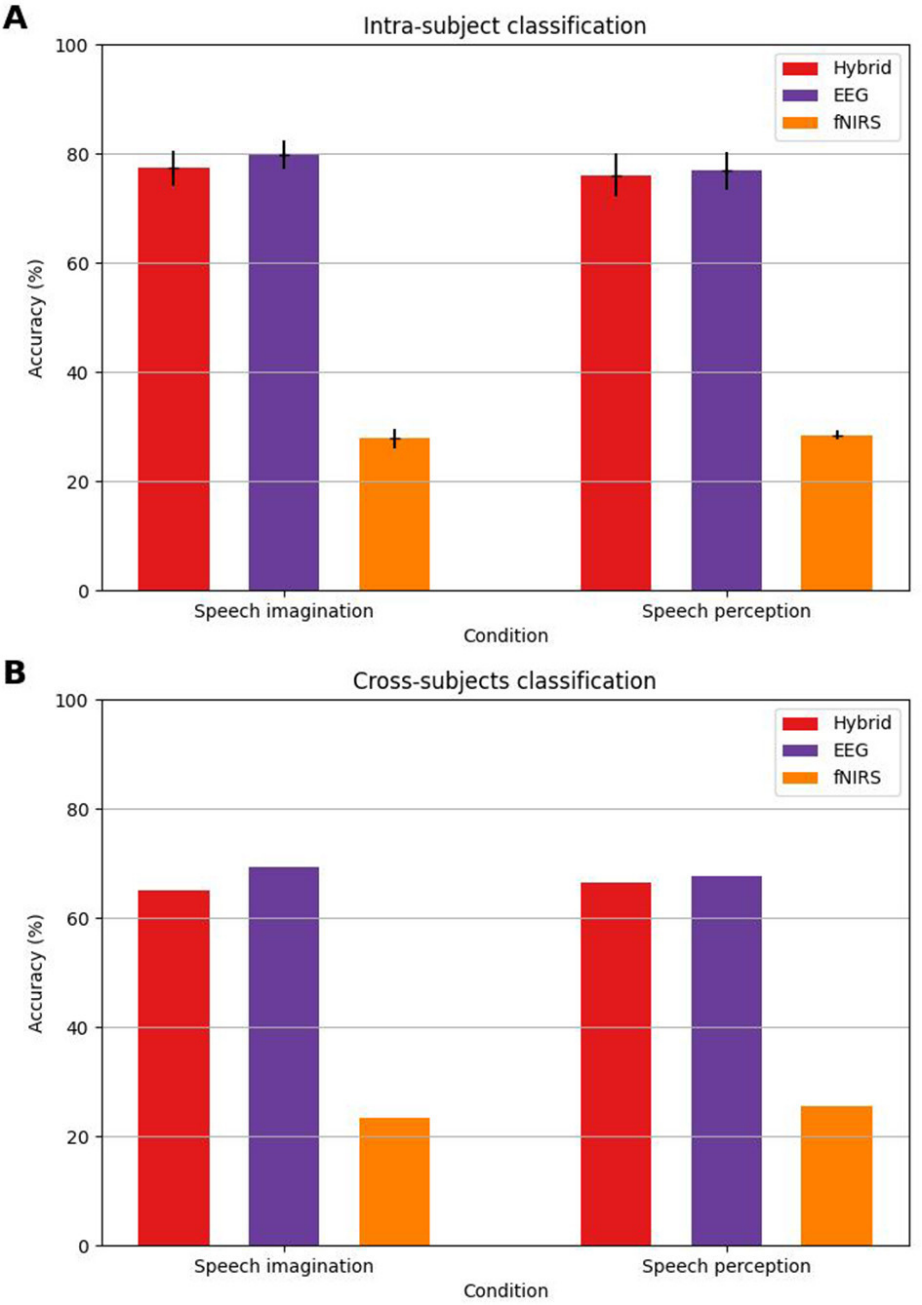
Classification accuracies of main classification protocols. Error bars indicate standard errors. **(A)** Intra-subject classification. **(B)** Cross-subjects classification.

**Figure 5:**
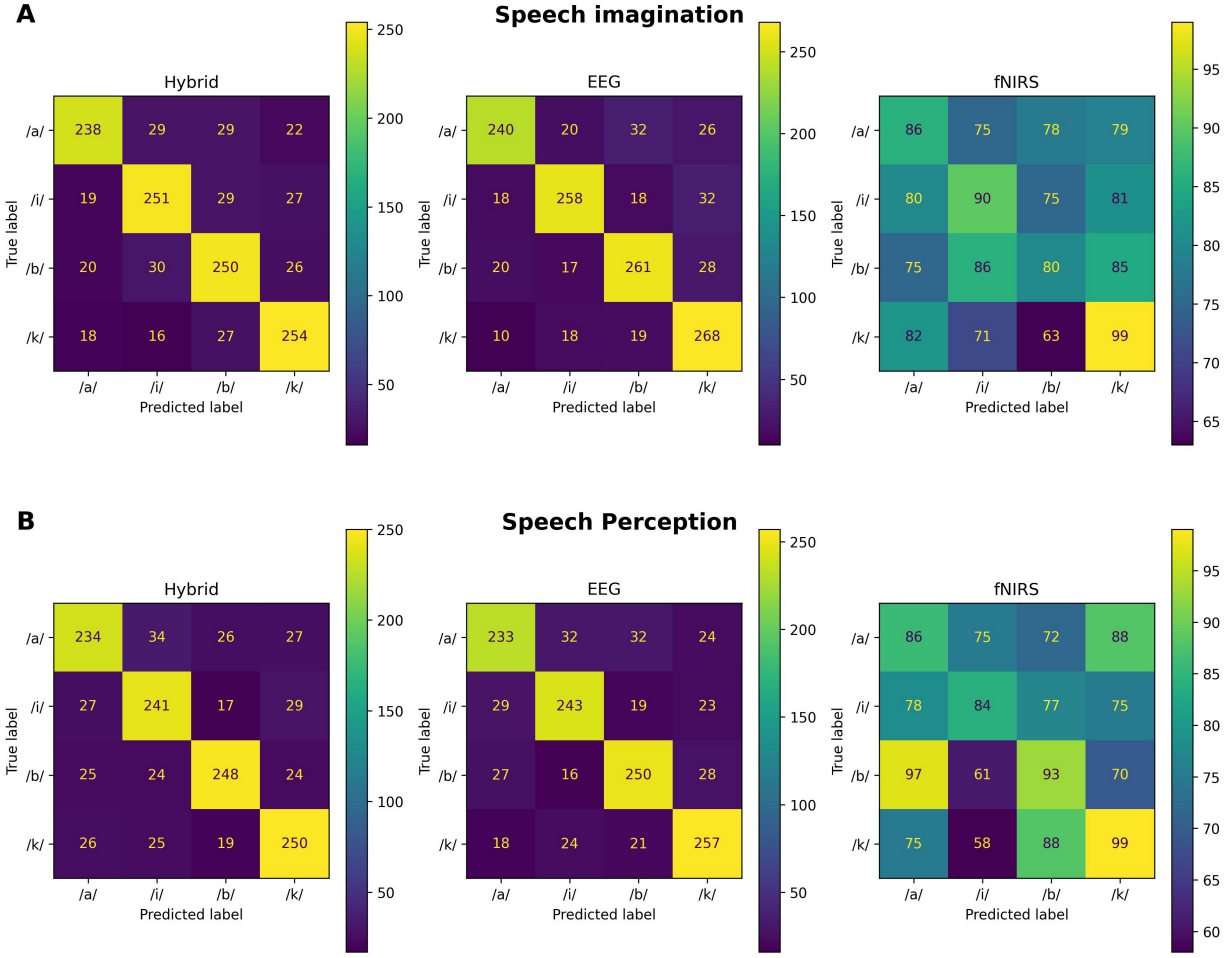
Confusion matrices of main classification protocols. **(A)** Imagined speech. **(B)** Perceived speech.

Both the individual and the cross-subjects classification results are summarized in Table 2 and visualized in Figure 4. Confusion matrices of intra-subject classification protocols are illustrated in Figure 5 and corresponding precision, recall and F1-scores are displayed in Table 3.

**Table 2:** Individual and cross-subjects classification accuracies (in %) achieved by the method with the feature selection procedure.

	**Imagination**			**Perception**		
**Participant**	**Hybrid**	**EEG**	**fNIRS**	**Hybrid**	**EEG**	**fNIRS**
P03	85.25	86.89	21.31	96.83	96.83	30.16
P04	90.16	90.16	18.03	93.55	93.55	29.03
P05	90.91	90.91	45.45	79.25	79.25	39.62
P06	44.07	59.32	20.34	37.93	41.38	32.76
P07	92.98	94.74	26.32	92.86	92.86	28.57
P08	79.66	84.75	35.59	60.00	53.33	33.33
P10	74.51	58.82	23.53	86.54	86.54	26.92
P11	70.97	83.87	30.65	55.74	68.85	29.51
P12	65.45	65.45	27.27	53.85	61.54	26.92
P13	80.65	79.03	24.19	60.00	68.33	23.33
P14	71.19	67.80	33.90	73.77	72.13	29.51
P15	51.79	60.71	30.36	75.93	72.22	27.78
P16	81.25	82.81	29.69	98.44	98.44	23.44
P17	70.49	81.97	37.70	89.66	89.66	36.21
P18	69.49	69.49	22.03	58.33	58.33	23.33
P19	89.09	90.91	27.27	71.93	71.93	28.07
P20	92.98	96.49	19.30	94.92	94.92	25.42
P21	66.67	79.37	23.81	81.25	81.25	26.56
P22	96.67	95.00	21.67	94.83	94.83	27.59
P24	86.79	81.13	47.17	88.24	80.39	27.45
P26	54.39	61.40	22.81	44.44	48.15	29.63
P27	94.92	93.22	22.03	84.75	84.75	20.34
*M*	77.29	79.74	27.75	76.05	76.79	28.43
*SD*	14.43	12.42	7.82	17.83	16.27	4.25
Cross subjects	64.97	69.36	23.43	66.35	67.69	25.59

**Table 3:** Precision, recall and F1-scores of the main intra-subject classification protocols.

**Classification protocol**	**Metric**	**/a/**	**/i/**	**/b/**	**/k/**
Hybrid imagination	Precision	80.68	76.99	74.63	77.20
	Recall	74.84	76.99	76.69	80.63
	F1-Score	77.65	76.99	75.64	78.88
EEG imagination	Precision	83.33	82.43	79.09	75.71
	Recall	75.47	79.14	80.06	85.08
	F1-Score	79.21	80.75	79.57	80.12
fNIRS imagination	Precision	26.63	27.95	27.03	28.78
	Recall	27.04	27.61	24.54	31.43
	F1-Score	26.83	27.78	25.72	30.05
Hybrid perception	Precision	75.00	74.38	80.00	75.76
	Recall	72.90	76.75	77.26	78.13
	F1-Score	73.93	75.55	78.61	76.92
EEG perception	Precision	75.90	77.14	77.64	77.41
	Recall	72.59	77.39	77.88	80.31
	F1-Score	74.20	77.27	77.76	78.83
fNIRS perception	Precision	25.60	30.22	28.18	29.82
	Recall	26.79	26.75	28.97	30.94
	F1-Score	26.18	28.38	28.57	30.37

On average, hybrid individual classification based on the proposed feature selection method correctly identified 77.29% (*SD* = 14.43) of imagined phonemes. In comparison, accuracy scores of 79.74% (*SD* = 12.42) and 27.75% (*SD* = 7.82) were achieved regarding unimodal EEG and fNIRS data, respectively. Both the hybrid (*t*_21_ = 13.71, *p* < 0.001) and the unimodal EEG version (*t*_21_ = 15.66, *p* < 0.001) exhibited a significantly higher classification accuracy than the unimodal fNIRS variant. Between hybrid and EEG classification no significant difference arose (*t*_21_ = 1.64, *p* = 0.12). All three classification protocols significantly exceeded chance level (hybrid: 95% CI [72.20, 82.23], *p* < 0.05; EEG: 95% CI [75.28, 84.02], *p* < 0.05; NIRS: 95% CI [25.09, 30.60], *p* < 0.05).

**Figure 6:**
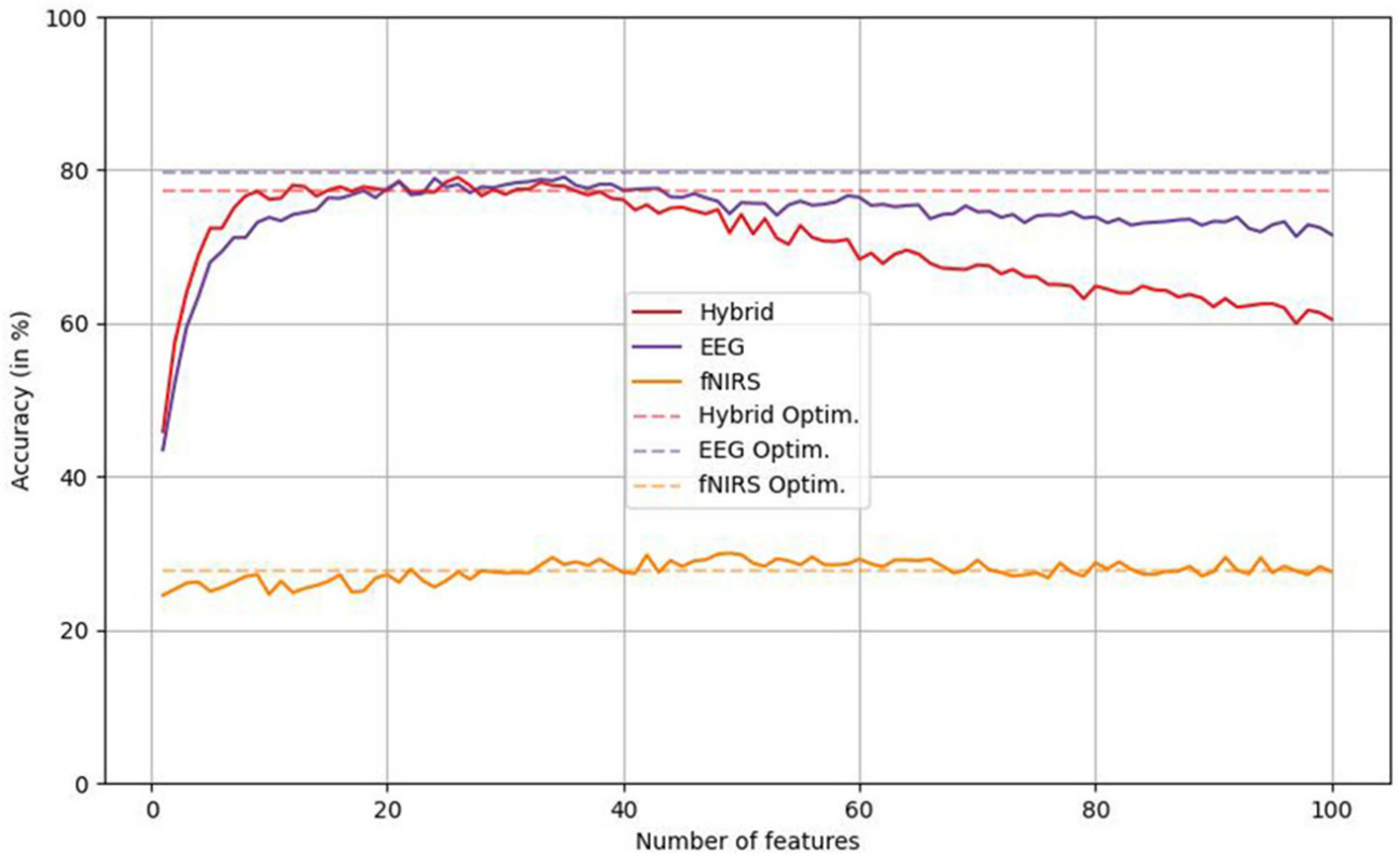
Classification performances over *k* = 0, 1, … 99, 100 features regarding hybrid, unimodal EEG- and unimodal fNIRS-data. Dashed lines illustrate the accuracy achieved by the respective parameter optimization protocol. Optim. = classification accuracies with feature number optimization.

The analysis of classification results based on *k* features for each *k* in 1, 2 … 99, 100 (Figure 6) revealed a plateau from approximately 10 to 50 features with a subsequent drop-off regarding both hybrid and unimodal EEG classifications, but more pronounced for the former. Classification performance based on fNIRS data showed a slight improvement as a function of number of features. When employing the proposed feature selection method, the hybrid protocol outperforms 83 of 100 fixed *k* classifications, unimodal EEG classification outperforms all 100, and unimodal fNIRS-based classification outperforms 53.

When running the main classification protocol (intra-subject imagined speech classifications with the proposed feature selection mechanisms) with beta-coefficients as fNIRS features, a hybrid accuracy of 80.16% (*SD* = 12.81). Despite achieving an average improvement of 2.87%, hybrid classification based on beta-coefficients as fNIRS features did not perform significantly better than with standard fNIRS features (*t*_21_ = 1.73, *p* = 0.10). Unimodal fNIRS classification effected an accuracy score of 27.80% (*SD* = 5.80), which did not significantly differ from fNIRS classification performance with standard features (*t*_21_ = 0.03, *p* = 0.98).

The results concerning the classification of heard phonemes are summarized in Table 2: hybrid and unimodal EEG phoneme discrimination procedures yielded accuracy scores of 76.05% (*SD* = 17.83) and 76.79% (*SD* = 16.27), respectively, while fNIRS based classification exhibited an accuracy of 28.43% (*SD* = 4.25). Hybrid and unimodal EEG classification did not differ significantly (*t*_21_ = 0.77, *p* = 0.45), while both significantly outperformed fNIRS classification (Hybrid: *t*_21_ = 11.16, *p* < 0.001; EEG: *t*_21_ = 11.96, *p* < 0.001). All three classification protocols significantly exceeded chance level (hybrid: 95% CI [69.58, 82.00], *p* < 0.05; EEG: 95% CI [70.99, 82.44], *p* < 0.05; NIRS: 95% CI [26.97, 29.96], *p* < 0.05).

Classifications based on the combined data of all participants yielded the following results: Concerning imagined speech, the hybrid approach correctly identified 64.97% of phonemes, the EEG-based approach 69.36% and the fNIRS-based approach 23.43%. Regarding heard phonemes, hybrid, EEG-based, and fNIRS-based classification achieved accuracy scores of 66.35%, 67.69% and 25.59%, respectively.

### 3.1 The contribution of feature types to hybrid imagined speech classification

**Figure 7:**
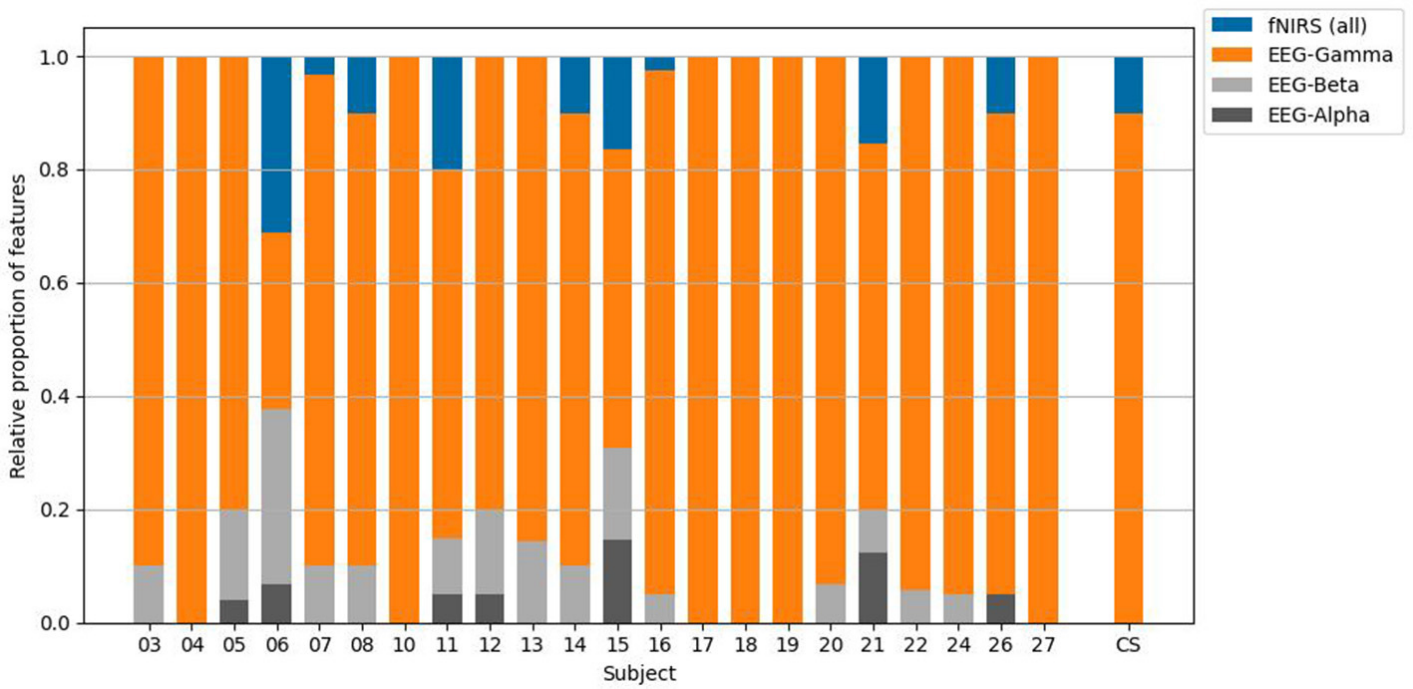
The relative proportions of recruited feature types. CS = cross-subjects.

Figure 7 illustrates the contribution of each neuroimaging modality to the individual hybrid imagined speech classification procedure, i.e., the percentage of features selected by the mutual information algorithm. As becomes evident, the EEG modality dominated the classification process, as gamma power features accounted for 84.36%, beta features for 7.86%, alpha features for 2.39% and fNIRS based features for 5.40%. Regarding the consolidated data of all participants, the selected feature set consisted of EEG gamma power (90.00%) and fNIRS features (10.00%), exclusively.

### 3.2 The contribution of feature types to unimodal imagined speech classification

**Figure 8:**
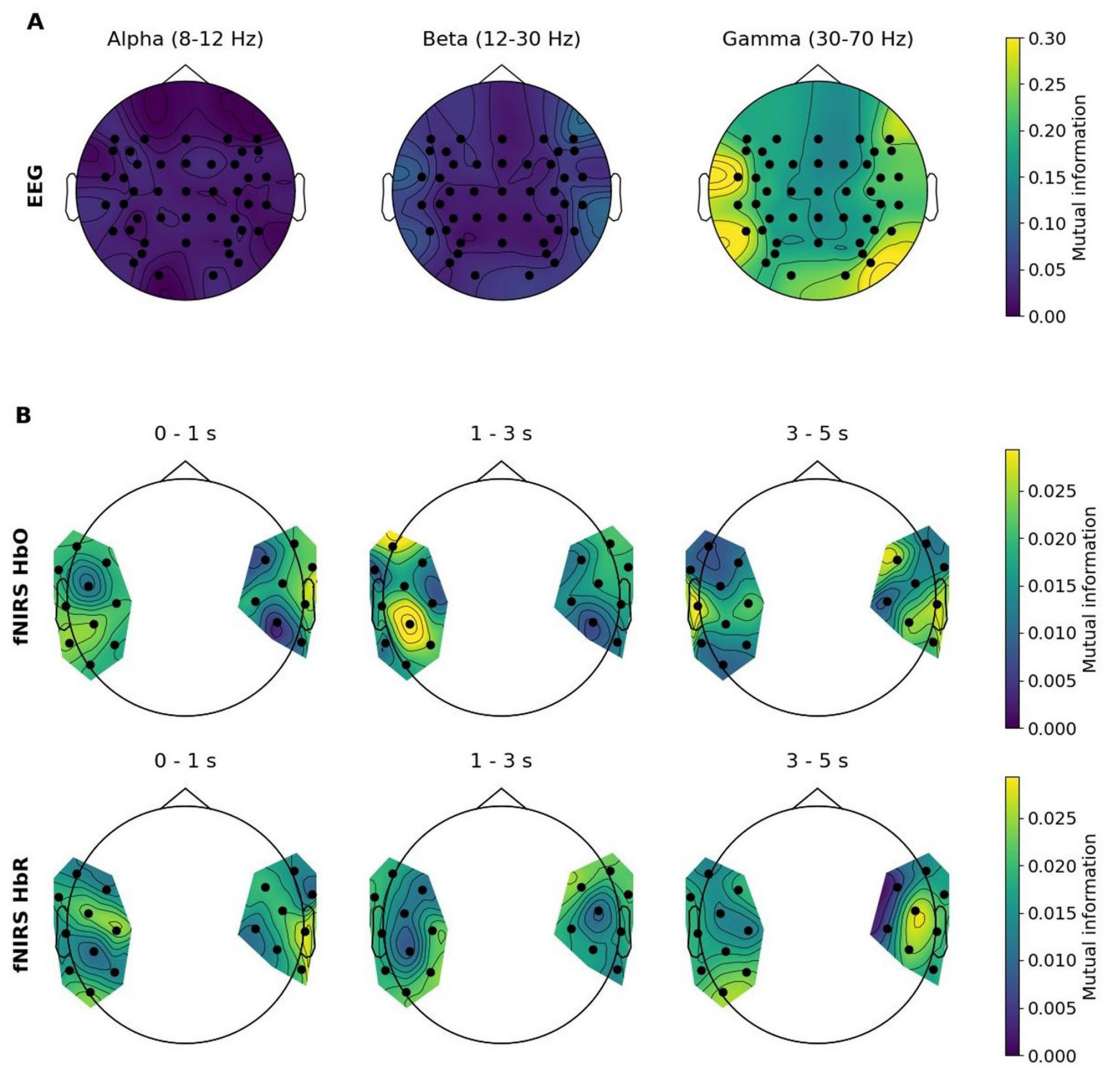
Topographic maps of average MI values across all participants for each feature type. **(A)** EEG feature types: Power spectral densities of the alpha, beta and gamma frequency bands. **(B)** fNIRS feature types: Oxy-hemoglobin (HbO) and deoxy-hemoglobin (HbR) concentration changes in three different time windows: 0-1s, 1-3s and 3-5s after trial start.

**Figure 9:**
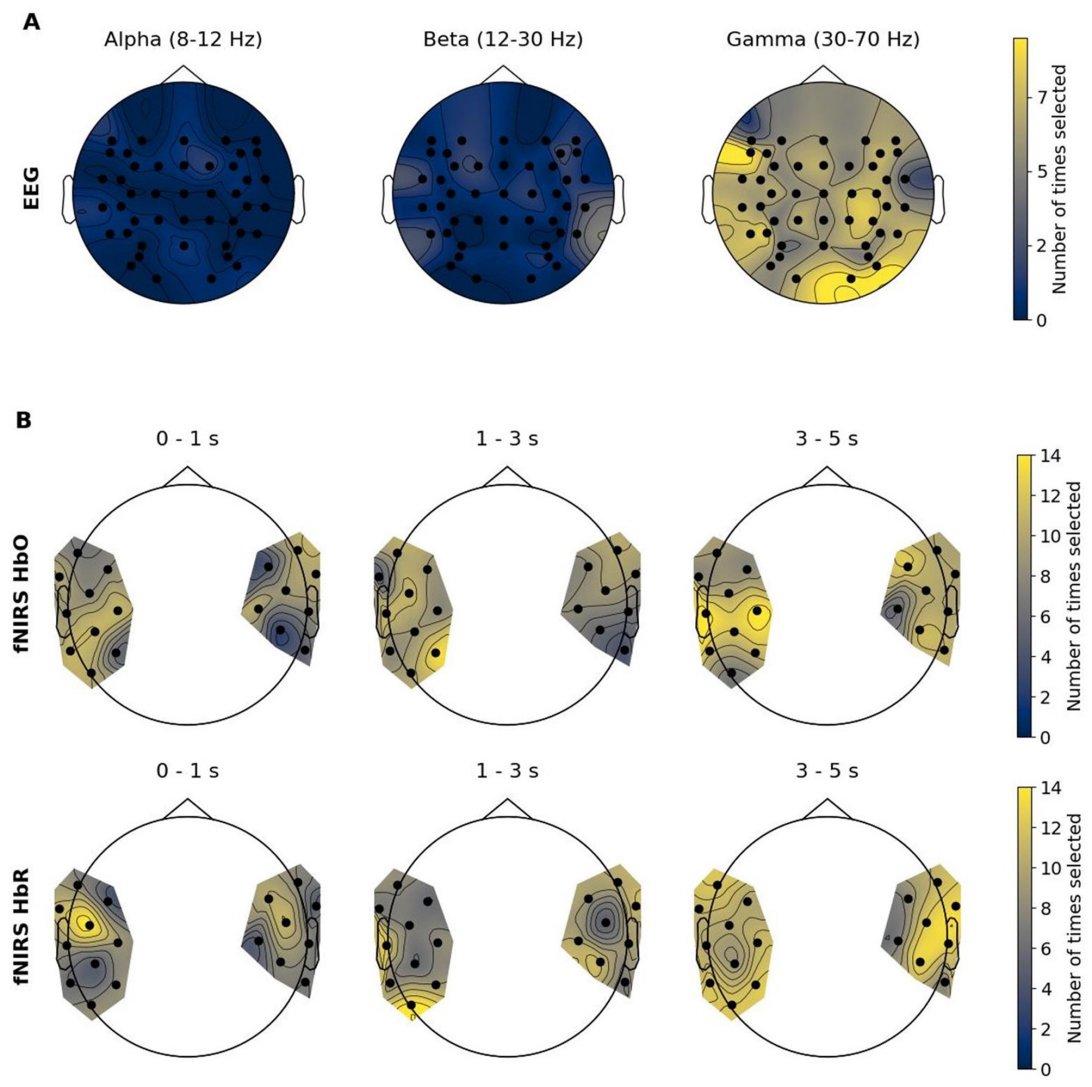
Topographic maps of the number of times each feature has been included in the selected feature set. **(A)** EEG feature types: Power spectral densities of the alpha, beta and gamma frequency bands. **(B)** fNIRS feature types: Oxy-hemoglobin (HbO) and deoxy-hemoglobin (HbR) concentration changes in three different time windows: 0-1s, 1-3s and 3-5s after trial start.

Figure 8 depicts topographic maps representing MI values of each EEG frequency range as well as fNIRS time windows for both chromophores averaged over all participants with respect to imagined speech classification. Figure 9 illustrates how frequently features were recruited for the classification. As a reminder, features that were used for classification were selected based on the MI coefficient. Features holding higher MI values signal stronger dependencies on the target vector, while coefficients approximating zero indicate no relationship between variables. Regarding the EEG modality, it becomes evident that gamma features showed higher MI than beta, and beta features showed higher MI than alpha features, on average. In general, fronto-temporal brain areas showed higher MI values, especially in comparison with centro-parietal ones. Furthermore, a right occipital pole emerged. The topomaps in Figure 9 show a similar mechanism: gamma features were selected more frequently than beta and alpha features, and beta features were selected more frequently than alpha features. Right occipital, right fronto-central and left temporal areas were recruited most frequently. Due to the low classification performance in the fNIRS domain, the topoplots are not analyzed here. They are solely provided for the purpose of entirety and comprehensiveness.

## 4 Discussion

The purpose of the present study was to discriminate four phonemes /a/,/i/,/b/ and /k/ from neural data by means of MLPs. Phonemes were either imagined or auditorily perceived, and brain signals were recorded using electrophysiological data derived from EEG as well as hemodynamic signals using fNIRS. The primary focus was to investigate the classification performance regarding imagined speech based on hybrid EEG-fNIRS data. With an accuracy score of 77.29%, the proposed method surpassed the a priori defined minimum requirement of 70% in terms of classification performance (Kübler et al., 2006). The hybrid classification protocol did not differ significantly from the unimodal EEG-based classification (79.74%), while the fNIRS-based classification scheme performed significantly worse (27.75%).

### 4.1 The benefit of the proposed feature selection technique

To assess the reasonability of the MI-based feature selection technique employed in the current study with respect to imagined speech classification, we incorporated a baseline protocol: When comparing the proposed feature selection method with “blindly” selecting the *k* most discriminative features with *k* = 1, 2 … 99, 100, the hybrid and the EEG classification variant outperformed classifications across 83 and all *k*, respectively, while the fNIRS variant outperformed classifications across 53 *k*.

This is consonant with the findings of Deligani et al. (2021), who evaluated a mutual information based feature selection technique and compared it to a classification based on all features. The feature selection method increased hybrid EEG-fNIRS based classification accuracy regarding the discrimination of an ALS group and a healthy control group using a visuo-mental task by 16.67%. Similarly, Guerrero-Mosquera et al. (2010) also showed that their mutual information based feature selection technique performed better than incorporating all available features of a fivefold seizure type classification problem. The benefit, however, was less pronounced, yielding a 1% increase as compared to the classification based on all features.

Generally, the proposed feature selection method was beneficial even when little discriminative information was present in the data. Further, the feature selection technique appeared to be most favorable for more complex data, which corresponds to the hybrid feature set in our case.

### 4.2 Performance comparison to previous imagined speech classification studies

To this date, only few studies engaged in the classification of imagined speech elements from hybrid EEG-fNIRS data. Rezazadeh Sereshkeh et al. (2019b), for instance, were able to distinguish between “yes”, “no” and rest classes with a ternary classification accuracy of 70.45%. Another group achieved an average fourfold accuracy score of 34.29% by classifying either four action words or four adjective-noun compounds (Cooney et al., 2022). Within this small fraction of BCI literature the hybrid classification protocol of the present study yielded the best performance so far, with performance improvements of 8.52% and 44.68%, respectively. Note, however, that comparisons are limited due to the diversity in linguistic stimuli, algorithms, trial duration, number of features etc.

EEG accounts for the vast majority of imagined speech classification literature. Matsumoto and Hori (2014), for example, attempted to classify five imagined vowels /a/,/i/,/u/,/e/ and /o/ by using SVMs. They extracted common spatial patterns from each channel and thereby yielded a classification accuracy of 79%. However, exclusively pairwise vowel discrimination was performed. One of the first studies to attempt imagined vowel discrimination was conducted by DaSalla et al. (2009). They computed common spatial patterns of/a/and/u/trials and achieved a binary classification accuracy of 62.66% by using an SVM. A more recent study (Chengaiyan et al., 2020) performed classifications of vowels a, e, i, o and u. However, binary classifications were performed by contrasting vowels with their negations in a one-versus-rest manner, e.g, “a” vs. not “a”. They extracted brain connectivity estimators from EEG and achieved a classification accuracy of 80% by using a deep belief network (DBN). In another recent study, vowels a, i and u were decoded with a ternary classification accuracy of 83.78% (Panachakel and Ganesan, 2021). They extracted magnitude squared coherence values from each EEG channel whereby a deep neural network was fed. Another group of researchers made use of deep neural networks and yielded an accuracy score of 85.66% with respect to the imagined vowels a, e, i, o and u (Sarmiento et al., 2021). A deep learning algorithm based on convolutional neural networks (CNNs) was used. Note that the authors trained 10 classifiers, each corresponding to a particular vowel pair, which were then combined in a voting scheme. By selection of the vowel with the most votes they obtained the final prediction.

Although some studies reported higher accuracies than the present study, classification was often conducted in a binary fashion. In contrast, fourfold classification of imagined phonemes was conducted in the present study, yielding an accuracy score of 77.29%. This not only exceeds the suggested threshold for practical BCI use of 70% (Kübler et al., 2006), but also represents a more transparent evaluation of the classifier's performance compared to binary decoding strategies for a multiclass problem. MLP classifiers were applied in the present study, which represent a more sophisticated algorithm than e.g., SVMs, but also require less computational cost and, consequently, less time during classification compared to modern deep learning techniques such as CNNs.

The given literature overview highlights the improvements of the present study made on multiclass imagined speech decoding, especially in comparison with previous hybrid imagined speech BCIs. However, these improvements were only observable in the hybrid and the unimodal EEG classification paradigms. While phoneme discrimination from EEG data yielded an average accuracy of 79.74%, only 27.75% of phonemes were correctly identified based on fNIRS data alone. In the following section this issue is elaborated on.

### 4.3 The contribution of fNIRS to hybrid classification

The classification accuracy achieved with unimodal functional near-infrared spectroscopy (fNIRS) was observed to be substantially inferior compared to both the hybrid and unimodal EEG approaches regarding both task conditions. This finding suggests that fNIRS data did not contribute significantly to the enhancement of hybrid classification models, offering limited discriminative utility when incorporated with EEG data. Contrary to previous work in the field, which suggested high effectiveness for both modalities when used in isolation (Shin et al., 2018; Rezazadeh Sereshkeh et al., 2019b; Hosni et al., 2022) the current study indicates that optimal unimodal performance may be a critical precondition for achieving synergistic effects in hybrid classification systems.

To optimize the balance between information transfer and assessment duration, trials of 5 seconds with intervals ranging from 2 to 4 seconds were administered, drawing on a methodological rationale presented by Cooney et al. (2022), who explored hybrid imagined speech classification with similarly brief trials and rest intervals. Despite the modest decoding performance of fNIRS (29.64% for a fourfold classification challenge), improvements over unimodal approaches were observed when the modalities were combined. Nevertheless, a potential limitation of trial count and duration was identified, given that Cooney et al. were restricted to a maximum of 50 trials per class per participant, a limitation caused by the COVID-19 pandemic and the exclusion of artifact-laden trials. There was an expectation that increasing the number of trials and their duration could enhance classification performance.

The present study also drew inspiration from earlier research by Chiarelli et al. (2018), whose hybrid motor imagery brain-computer interface (BCI) employed 5-second trial durations to achieve notably high accuracy rates for unilateral EEG and fNIRS classifications, with even greater success for their hybrid system. This reinforces the potential for synergistic effects with such trial durations. Preliminary recordings from a volunteer suggested that peak hemodynamic responses occurred within the 5-second interval, with Huppert et al. (2006) substantiating that the HbO response in fNIRS signals typically peaked around 4.6 seconds.

However, examination of individual, averaged fNIRS time courses related to the study conditions (Supplementary Figures 1, 2) revealed significant variability—the full nuances of the hemodynamic response function (HRF) were not universally reflected. A distinct HbO peak was not consistently evident across subjects and conditions within the prescribed 5-second window due to substantial heterogeneous response patterns. Despite this variability, nearly all participants exhibited chance-level classification scores. Evidence from Rezazadeh Sereshkeh et al. (2019a) demonstrated that variability in fNIRS responses does not preclude discriminability, as reflected in their satisfactory classification accuracies for imagined speech tasks.

The relatively brief inter-trial rest periods may have affected the discriminability of fNIRS data, as studies often deploy rest intervals exceeding 10 seconds to enable HRF recovery (Chiarelli et al., 2018; Rezazadeh Sereshkeh et al., 2019b; Guo and Chen, 2023). The efficiency of shorter rest intervals in hemodynamic measures is nonetheless documented, with previous robust trial investigations conducted with 2-second intervals (Dale and Buckner, 1997; Park and Rugg, 2008; Heilbronner and Münte, 2013; Schaeffer et al., 2014; Cooney et al., 2022). In our analysis, a downward trend was evident in averaged fNIRS data, hinting at pre-trial contamination—residual HRF from one trial mixing with the evoked response of the next. We attempted to mitigate this by employing classifications derived from beta coefficients, reflecting the fit of the empirical fNIRS signal to modeled canonical HRFs; however, this approach displayed lower classification acccuracy compared to standard fNIRS features.

In summary, fNIRS data did not contribute meaningfully to the hybrid execution of classifying imagined or perceived phonemes. The abbreviated trial and rest durations potentially precluded the capture of the complete temporal dynamics of the HRF, and moreover, the short rest intervals may have introduced interference from preceding trials that the beta-coefficient based classification approach failed to rectify.

### 4.4 The influence of different features and channels

Since fNIRS classification was characterized by low accuracies as well as low corresponding MI scores, only EEG features are subject of this chapter. In the present study, gamma frequency power showed clear dominance in recruited feature sets, followed by the beta and lastly the alpha band. This finding is consistent with other imagined speech studies which attributed a similar higher discrimination potential to beta and gamma compared to lower frequencies (Pei et al., 2011; Rezazadeh Sereshkeh et al., 2019b; Lee et al., 2020; Cooney et al., 2022; Preedapirat and Wongsawat, 2022). Regarding the neural correlates of imagined speech, research suggests the involvement of brain areas similar to those being active during speech execution (Panachakel and Ramakrishnan, 2021). According to Tian and Poeppel (2013) articulatory planning occurs in the inferior frontal gyrus (IFG) during speech imagery. Since no voluntary muscle movements are intended, information flow terminates at the primary motor cortex (M1). However, from M1 efference copies are first sent to the inferior parietal cortex, to the pSTG (Wernicke's area) and the superior temporal sulcus (STS). Additionally, the primary auditory cortex in the STG and the middle temporal cortex are considered to be involved in the retrieval of abstract auditory representations and episodic memory information, respectively. The results of the present study indicate partial conformity with this postulated process. When considering the channel positions with the highest corresponding MI values, the right IFG and the left temporal lobe provided the most discriminative information, on average. The influence of the IFG was expected to be more strongly pronounced on the left hemisphere rather than on the right, as literature suggests a role of Broca's area in imagined speech (Hesslow, 2002; Pei et al., 2011; Tian and Poeppel, 2013). However, evidence exists that supports the notion of a discriminative role of the right IFG in imagined speech. Rezazadeh Sereshkeh et al. (2019b), for example, found that the right IFG discriminated more strongly between “yes” and “no” imagined speech classes than its left-hemispheric homolog in the beta and alpha band. This is further supported by an fMRI study that found significant right-hemispheric IFG activation during first-person speech imagery (Shergill et al., 2001). The discriminative potential of the right IFG may be attributed to differences in fine motor control with respect to the phonemes (Liakakis et al., 2011). As speaking generally involves fine facial motor actions, and the phonemes used in the current study were selected with respect to their articulatory dissimilarities, this explanation is plausible. Surprisingly, PO8 also showed comparatively high MI scores and was one of the most frequently selected channels. Although involvement of occipital regions is not suggested in established hypotheses concerning the neural correlates of imagined speech, similar results were already reported elsewhere (Nieto et al., 2022). This finding is supported by an fMRI study conducted by Sugimori et al. (2014), who identified higher activations in bilateral occipital regions during imagined speech compared to perceived speech. However, caution is advised regarding the interpretation of this finding, as the discriminative capability of occipital brain activation may merely stem from differences in visual processing of the phonemes.

### 4.5 Perceived speech and cross-subjects classification

Concerning the perceived speech condition classification performance turned out slightly lower than the one observed regarding imagined speech. This finding is unexpected as classifying perceived speech units proved more successful than imagined speech according to existing literature (Martin et al., 2016; Sharon et al., 2020). It may well be that imagined speech entailed a better maintenance of attention, since it requires active participation in contrast to merely listening to spoken phonemes.

In addition to classifying based on individual data, cross-subjects decoding of phonemes was also attempted. The datasets subjected to this kind of analysis contained consolidated data of all participants. Using hybrid EEG-fNIRS-data, 64.97% of imagined phonemes were correctly categorized, while an accuracy score of 69.36% was achieved based on EEG data alone. This reveals substantial cross-subjects decoding of mentally spoken phonemes. Associated with a below-chance accuracy regarding unimodal fNIRS classification (23.43%), fNIRS features evidently hampered the overall performance of the hybrid phoneme classifier. Concerning the cross-subjects EEG classification of imagined speech, decoding performance surpassed the fivefold classification accuracy of 67.76% reported by Sarmiento et al. (2021). They aimed to classify five vowels by training 10 classifiers, one for each vowel pair. This represents a more computationally expensive method compared to the one proposed in this study and relies on binary classification, whereas the accuracy reported in the current study stemmed from a fourfold classification task. A similar trend emerged regarding auditorily perceived phonemes (hybrid: 66.35%, EEG: 77.69%, fNIRS: 25.59%). No previous studies were found that engaged in the cross-subjects decoding of perceived speech.

## 5 Limitations and future directions

In both conditions assessed, no significant differences in performance were observed between hybrid classification models and those solely based on EEG. The modest performance of classifications using fNIRS data is attributed to the truncated trial durations and rest intervals. Although this design choice was supported by a multitude of studies in the literature (Dale and Buckner, 1997; Park and Rugg, 2008; Heilbronner and Münte, 2013; Schaeffer et al., 2014), even in the hybrid imagined speech classification domain (Cooney et al., 2022), it was unable to capture the full hemodynamic signal course and likely led to pre-trial signal interference.

Moreover, the limited phonemic array employed in this study raises concerns regarding the generalizability of imagined speech brain-computer interfaces (BCIs) as pragmatic communication tools for individuals with motor disabilities. A comprehensive and consistent interpretation of a wider spectrum of letters or phonemes from cerebral activity is imperative for their utility. Furthermore, there is an abundance of research focusing solely on vowel discrimination (Matsumoto and Hori, 2014; Chengaiyan et al., 2020; Panachakel and Ganesan, 2021; Sarmiento et al., 2021). As the phonemes used in the current study were selected with respect to their phonetic and articulatory differences (Keating, 1984; Tronka, 2004), this evokes skepticism about the capability of the current classification schema to discriminate between, for example, the same number of vowels with comparable precision.

Drawing from the insights gathered, future research in hybrid BCI systems should consider extending classification windows to a minimum of 10 seconds paired with adequate inter-trial intervals when optimized decoding precision is desired and low temporal resolution can be tolerated. Conversely, when efficiency and pace are key objectives, building a unimodal classification framework based on EEG appears to be more advantageous than a hybrid pendant. In pursuit of the overarching goal to deliver effective and efficient communication prostheses for individuals with communication impairments, it is imperative that forthcoming studies in offline BCI classification not only pay attention to the inherent design features necessary for online deployment but also leverage computationally efficient yet potent machine learning frameworks and classification techniques. Finally, cross-participant decoding results should be systematically reported, promoting the advancement of universally applicable BCIs through the confirmation of class discrimination efficacy across combined datasets involving multiple subjects.

## 6 Conclusion

In the present study an offline classification system designed to decode mentally spoken and perceived phonemes from EEG and fNIRS signals was developed. Both hybrid and EEG based imagined phoneme classification yielded good performance (> 75%). These classification procedures outperformed previous comparable classification studies. Phoneme discrimination based on fNIRS data alone exhibited a comparably low but higher-than-chance-level accuracy. Overall, similar results were observed regarding perceived speech. While both hybrid and unimodal EEG-based (>60%) cross-subjects classification produced surprisingly good accuracies, intra-subject phoneme discrimination generally performed better. Finally, the individual MI-based feature selection technique was beneficial, as indicated by comparisons with classifications based on k = 1, 2, … 99, 100 features selected by mutual information.

## Data Availability

The datasets presented in this study can be found in online repositories. The names of the repository/repositories and accession number(s) can be found below: Hybrid EEG-fNIRS phoneme classification based on imagined and perceived speech: https://osf.io/f7rq2.
