# Supplementary figures and images for: Hybrid EEG-fNIRS phoneme classification based on imagined and perceived speech
Source: Front Neuroergon. 2026 Feb 10;7:1696865. doi: 10.3389/fnrgo.2026.1696865 (PMC12929534; doi:10.3389/fnrgo.2026.1696865)

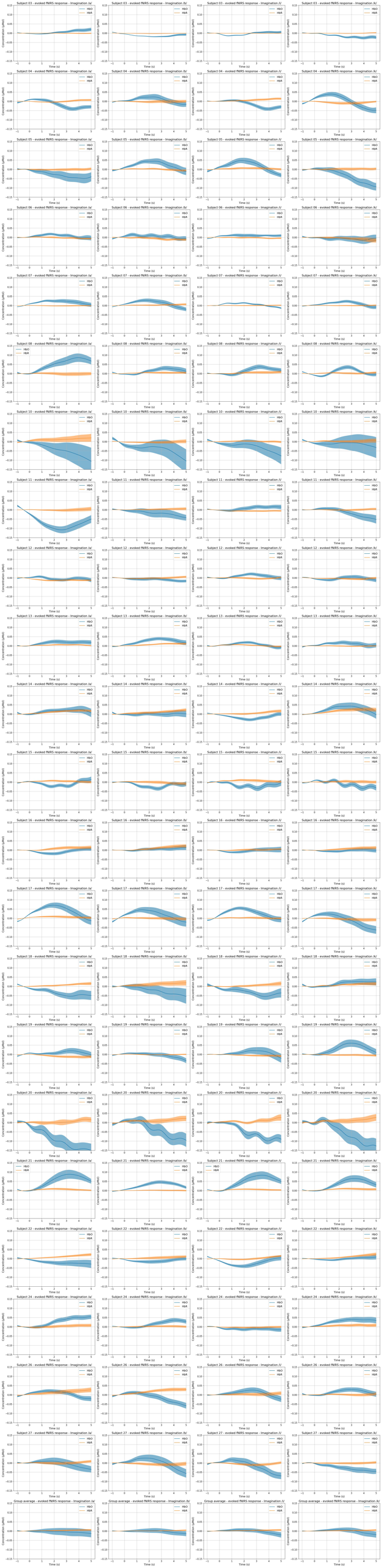

Supplement: Supplementary file 1 [file Image_1.jpeg]

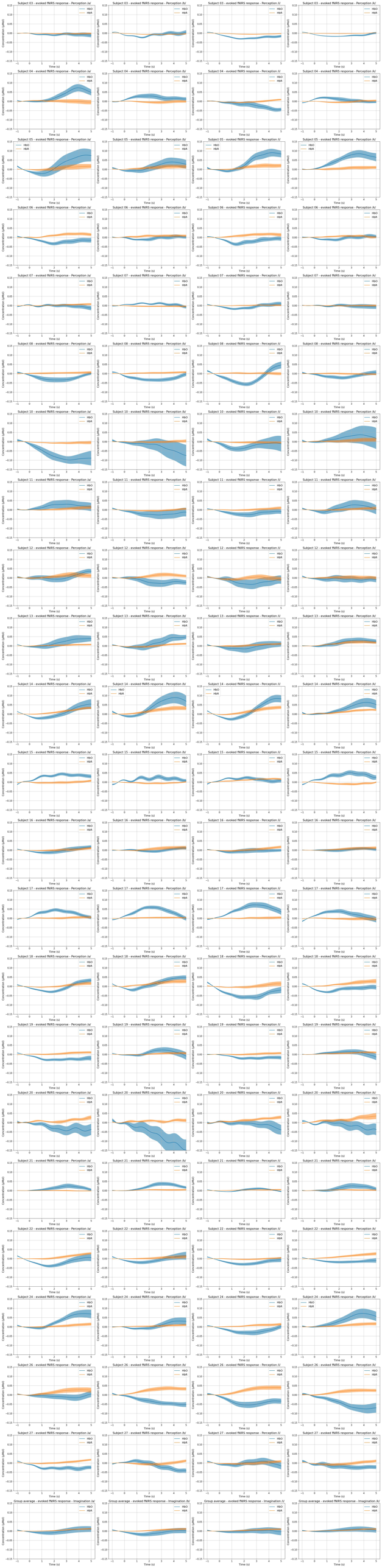

Supplement: Supplementary file 2 [file Image_2.jpeg]
